# Factors Associated with Attempt for Smoking Cessation among Hardcore Smokers in Taiwan

**DOI:** 10.31372/20200504.1117

**Published:** 2021

**Authors:** Hui-Wen Huang, Ya-Hui Yang, Wen-Wen Li, Chih-Ling Huang

**Affiliations:** a ESH Integration Department, Innolux Corporation, Tainan City, Taiwan; b Department of Nursing, Jianan Psychiatric Center, Ministry of Health and Welfare, Taiwan; c School of Nursing, San Francisco State University, California, United States; d School of Nursing, Fooyin University, Taiwan

**Keywords:** hardcore smokers, smoking cessation, attitudes towards the Tobacco Hazards Prevention Act, factor

## Abstract

**Background:** Tobacco control activities have mostly influenced those smokers who found it easier to quit and, thus, remaining smokers are those who are less likely to stop smoking. This phenomenon is called “hardening hypothesis,” which individuals unwilling or unable to quit smoking and likely to remain so. The aim of this study was to identify the factors correlated with smoking cessation among hardcore smokers.

**Methods:** A cross-sectional descriptive correlational research design was employed. Hardcore smokers from communities in Taiwan were recruited to participate in the study (*N* = 187). Self-report questionnaires were used to collect demographic data as well as data on nicotine dependence, quitting self-efficacy, social smoking motives, attitudes towards the Tobacco Hazards Prevention Act (THPA), and smoking cessation. Logistic regression analysis was used to examine the factors that were related to quit smoking.

**Results:** About 30.3% (*n* = 54) reported having experienced quitting smoking over 7 days in the past year. Logistic regression analysis indicated that attitudes towards the THPA was identified as a particularly important factor contributing to the increase in smoking cessation among hardcore smokers.

**Conclusions:** Nurses should cooperate with smoking cessation coaches to facilitate the improvement of attitudes towards the THPA as a key means through which to increase the smoking cessation rate among hardcore smokers.

## Introduction

Smoking is a major risk factor for chronic diseases and cancer (World Health Organization, 2019). The prevalence of smoking among adults in Taiwan, 29.2% in 1997 and 15.4% in 2017, was gradually decreasing due to the Tobacco Hazards Prevention Act (THPA) (Health Promotion Administration, 2019). However, the smoking rate has stopped decreasing among adults in Taiwan and the United States in recent years (Centers for Disease Control and Prevention, 2018; Health Promotion Administration, 2019). From a theoretical perspective, the prevalence of adult smoking tends to plateau following a period of considerable reduction in the pervasiveness thereof. This phenomenon, termed “hardening,” postulates that there might exist daily and long-term smokers who are unwilling to quit as hardcore smokers (Warner & Burns, 2003).

The Transtheoretical Model (TTM) was applied to the process of smoking cessation in a previous study (Kumar, Tiwari, Gadiyar, Gaunkar, & Kamat, 2018). According to the TTM, behavior modification occurs via a progression through five stages of change: (1) precontemplation: individuals do not intend to take action in the next 6 months; (2) contemplation: individuals examine the impact and consequences of their behavior and begin to formulate an intention to take action in the next 6 months; (3) preparation: individuals make a commitment to changing their behavior and develop a plan to take action in the immediate future; (4) action: individuals put their plan into action; and (5) maintenance: individuals make and sustain specific overt modifications to their lifestyles (Prochaska, DiClemente, & Norcross, 1992). In previous studies, hardcore smokers were defined as those not planning to quit smoking within the next 6 months, with knowledge of the risks of smoking, and continuing to smoke during the course of the study (Huang, Hsueh, Li, & Huang, 2020; Kang, Lee, & Cho, 2017). Thus, on the basis of TTM, hardcore smokers were in the precontemplation stage. According to TTM, the stages from smoking to cessation behavior are back and forth. Therefore, in this study, the outcome variable is the attempt behavior of smoking cessation in the past year. In order to motivate the hardcore smokers to change their smoking behaviors, the influencing factors of trial cessation behavior need to be examined.

Previous studies have estimated that the prevalence of hardcore smokers ranged from 19.7% to 29.4% (Azagba, 2015; Park et al., 2020). It is difficult for nurses or health clinicians to convince hardcore smokers to quit smoking. Therefore, understanding the factors related to smoking cessation in hardcore smokers is important for nurses or health clinicians who can design programs to encourage hardcore smokers to reduce their consumption or quit smoking altogether.

Previous studies only examined characteristics associated with individuals becoming hardcore smokers rather than smoking cessation among hardcore smokers. Older age, fewer years of education, and greater total number of years smoked increased the risk of becoming hardcore smokers (Bommele et al., 2015; Kaleta, Usidame, Dziankowska-Zaborszczyk, Makowiec-Dąbrowska, & Leinsalu, 2014). Fewer studies have examined the association between demographic characteristics and smoking cessation. Therefore, age, total number of years smoked, and years of education could be considered covariates in the examination of factors associated with smoking cessation among hardcore smokers.

Individual psychological factors, such as nicotine dependence and quitting self-efficacy, may be associated with smoking cessation. Nicotine dependence was defined as the urge to continue smoking and difficulty in smoking cessation (Benowitz, 2010). Hardcore smokers with greater nicotine dependence were more likely to experience nicotine withdrawal (Kawai et al., 2017). Quitting self-efficacy was defined as the confidence to resist smoking in high-risk smoking environments (Etter, Bergman, Humair, & Perneger, 2000). Bommele et al. (2015) found that hardcore smokers with higher quitting self-efficacy were more likely to quit smoking.

In Asian countries, such as Taiwan, people like to give cigarettes to and share cigarettes with others at social occasions as a common social custom. Consequently, nurses and researchers developed the scale of Social Smoking Motives which can be used to examine the association between social custom factors and smoking cessation (Huang, Cheng, & Huang, 2017). A previous study investigated smoking cessation among social smokers and nonsocial smokers, finding that social smokers were less likely to have the intention to quit smoking than were nonsocial smokers (Cai et al., 2017). Furthermore, smokers with a greater intention to quit were more likely to quit successfully (Smit, Hoving, Schelleman-Offermans, West, & de Vries, 2014). However, comparatively less is known about the role of social smoking motives in smoking cessation among hardcore smokers.

THPA is an essential regulation for forbidding smoking. Hardcore smokers were less likely to comply with the THPA than were non-hardcore smokers (H. W. Huang et al., 2020), and it seems that hardcore smoker did not quit smoking regardless. This suggests that attitudes towards the THPA may be an important factor in smoking cessation among hardcore smokers. Despite this, few studies have examined attitudes towards the THPA as a factor associated with smoking cessation among hardcore smokers.

The purpose of this study was to examine the factors contributing to attempt to cease smoking among hardcore smokers in Taiwan. Previous studies indicated that nicotine dependence and quitting self-efficacy were significantly associated with smoking cessation (Bommele et al., 2015; Kawai et al., 2017). However, older age, fewer years of education, greater total number of years smoked, social smoking motives, and attitudes towards the THPA significantly increased the risk of becoming hardcore smokers. Few studies examined the association between these factors and smoking cessation among hardcore smokers. On the basis of our review of the literature, we hypothesized that age, education, total number of years smoked, nicotine dependence, quitting self-efficacy, social smoking motives, and attitudes towards the THPA were associated with trial smoking cessation in this group.

## Materials and Methods

### Design and Sample

A cross-sectional descriptive correlational study was conducted. A purposive sampling design was used to recruit participants. The inclusion criteria for participation were (a) age ≥ 20 years, (b) living in a community, (c) having no intention to quit smoking in the next 6 months, and (d) continuing to smoke despite the associated health consequences. The exclusion criteria were (a) possessing any severe communication problems, (b) having a diagnosed mental disorder, and (c) being a drug abuser. A total of 199 participants were obtained. Due to the low percent female smokers (2.4%) in the overall female population in Taiwan (Health Promotion Administration, 2019), this study excluded 21 female smokers. The final sample was composed of 178 male smokers. A power analysis determined that a sample of 178 male smokers would be adequate for bivariate correlation (Polit & Beck, 2017), based on effect size 0.33, an alpha level of 0.05, and power of 0.08.

### Instruments

#### Demographic Data

Demographic data collected included age, education, and age at first cigarette.

#### Attempt for Smoking Cessation

Attempt for smoking cessation was assessed by a question regarding the 7-day point prevalence of abstinence in the past year (Health Promotion Administration, 2019). Answers were coded as 0 = no and 1 = yes.

**Figure 1:**
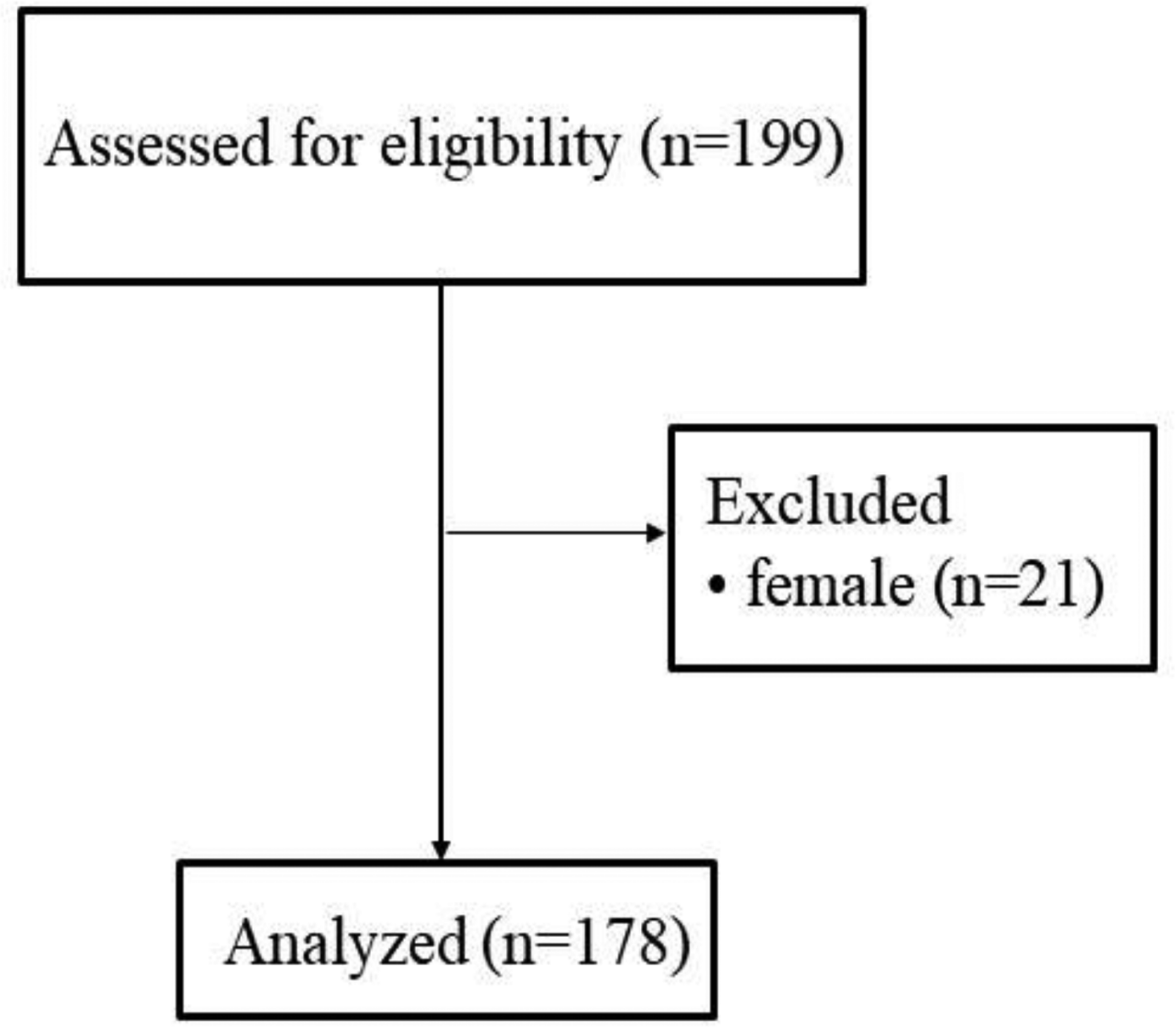
Data collection procedure.

#### Nicotine Dependence

A 6-item Chinese version of the Fagerstrom Test for Nicotine Dependence was used to measure the levels of degree of physical dependence on tobacco (C. L. Huang et al., 2006). The scale included two measuring morning smoking habits and four measuring daytime smoking habits. The range of total possible scores was 0–10, with higher scores indicating greater nicotine dependence. This study yielded an internal consistency reliability coefficient (Cronbach’s of 0.80).

#### Quitting Self-Efficacy

A 10-item Chinese-language version of the Smoking Self-Efficacy Questionnaire was used to measure participants’ confidence in their ability to resist the urge to smoke in a high-risk smoking situation (Etter et al., 2000). Each item was rated from “strongly disagree” (1 point) to “strongly agree” (5 points), with a possible total score ranging from 10 to 50; higher scores indicated that respondents had greater quitting self-efficacy. This study yielded a Cronbach’s *α* of 0.83.

#### Social Smoking Motives

The 12-item Chinese-language version of the Social Smoking Motives Scale, designed to assess social confidence and perceived social connection through smoking, was used to measure the smoking motives of participants (C. L. Huang et al., 2017). Each item was rated from “strongly disagree” (1 point) to “strongly agree” (5 points), with a possible total score ranging from 12 to 60; higher scores indicated that respondents had greater social smoking motives. The Cronbach’s *α* obtained in this study was 0.91.

#### Attitudes Towards the THPA

Attitudes towards the THPA were assessed to explore the perception of smoking policy in Taiwan. Ten items were included that measured attitudes towards the THPA, including: “Smoking is one’s own habit, so the government should not limit,” “Smoking policy is unfair and contradiction,” “Smokers who pay health surcharge of tobacco is ripped-off by government,” “If the government prohibit smoking, it should not sell cigarettes,” “Smokers are deprived of right by the smoking policies,” “Although the price of cigarettes was increased, I still continue to buy cigarettes,” “I continue to smoke regardless of the government’s encouragement of quitting/stopping smoking,” “I can deal with smoking policies,” “ I do not want to quit smoking regardless of the changes of smoking policies,” and “I would not go to the places where smoking is prohibited so I am able to smoke.” Each item was rated from “strongly agree” (1 point) to “strongly disagree” (5 points), with possible total scores ranging from 10 to 50, and higher scores indicating more positive attitudes to the THPA. This study yielded a Cronbach’s *α* of 0.82.

### Data Collection

Ethics approval for the design of the study was granted by the institutional review board of a medical center. Self-report questionnaires were administered to participants in communities. Nurses and a trained researcher described the purpose and process of the study to the participants and provided standardized directions to complete the questionnaires. Participants were informed that their answers on the study questionnaires would be treated anonymously, and that they could withdraw from the study at any time. Data were collected from December 2016 to May 2017.

### Data Analysis

The SPSS version 21.0 for Windows was used for data analysis. Descriptive analyses were used to describe participant characteristics. Chi-square tests and *t* tests were conducted to examine the differences between participants who had experiences of smoking cessation and those who did not. We controlled significantly different background factors between two groups as confounding factors in the logistic regression model. Logistic regression analysis was performed to identify factors that related to smoking cessation. A *p* value of less than 0.05 was interpreted as statistically significant for all tests.

## Results

### Descriptive Statistics of Factors

One hundred and seventy-eight hardcore smokers participated in this study. Of the participants, 90.4% were married and 77% had a job. Average age was 47.4 years, average age at first cigarette was 17.9 years, average number of cigarettes smoking per day was 17.0, average number of years of smoking was 29.4, and 30.3% of participants (*n* = 54) reported having quit smoking for over 7 days in the past year. The distributions of demographic characteristics, smoking background, quitting self-efficacy, and attitudes towards the THPA for participants were shown in Table 1. As indicated in Table 2, no statistically significant differences were found between participants with and without experience of smoking cessation for age, years of education, total number of years smoked, and social smoking motives. Nicotine dependence and quitting self-efficacy were significantly correlated with smoking cessation experience. The group with smoking cessation experience had significantly more positive attitudes on smoking policy than did the group without.

**Table 1:** Distributions of Demographic Characteristics, Smoking Background, Quitting Self-Efficacy, and Attitudes Towards the THPA for Participants

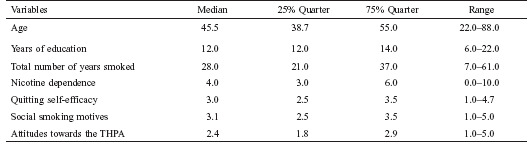

**Table 2:** Differences in Demographic Characteristics, Smoking Background, Quitting Self-Efficacy, and Attitudes Towards the THPA for Participants with Experience of Attempt for Smoking Cessation and those Without (N = 178)

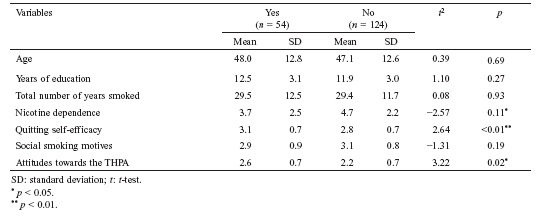

### Factors Associated with Smoking Cessation

Results indicated that attitudes towards the THPA was a statistically significant factor in smoking cessation among hardcore smokers, with more positive attitudes being associated with a higher likelihood of quitting (adjusted odds ratio [AOR = 1.80, 95% CI = 1.12–2.90]). Nicotine dependence, quitting self-efficacy, and social smoking motives were not statistically significant factors in smoking cessation among hardcore smokers (Table 3).

**Table 3:** Factors Associated with Attempt for Smoking Cessation among Hardcore Smokers (N = 178)

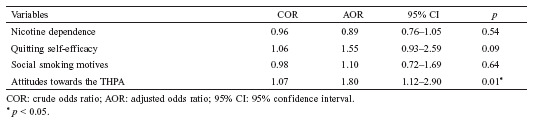

## Discussion

Few studies have reported the rate of smoking cessation among hardcore smokers. Only Lam, Cheung, Leung, Abdullah, and Chan (2015) found a 6-month quit rate among hardcore smokers of 12%. In this study, 30.3% of participants reported experiencing 7-day quit point in the previous year. It is possible that the discrepancy between their and ours findings may be due, in part, to a difference in the definition of smoking cessation. However, further research is required on the smoking cessation rates among hardcore smokers.

To our knowledge, ours was first study to examine the factors correlated with smoking cessation among hardcore smokers; we found that an important one of which was attitudes towards the THPA. Wu et al. (2015) also highlighted the impact of positive attitudes regarding the THPA on increasing levels of smoking cessation. Smokers with more positive attitudes towards the THPA were found to be more respectful of the law and had greater concern for others’ health (Chuang & Huang, 2012). Moreover, hardcore smokers with more positive attitudes regarding the THPA may comply with legislation mandating a smoke-free environment and may, thus, try to quit smoking. Having a better knowledge of the THPA is crucial to improving hardcore smokers’ attitudes thereto. Therefore, teaching knowledge of THPA may further assist in encouraging them to quit smoking.

The group with experience of smoking cessation had higher quitting self-efficacy than did the group without experience thereof in the univariate analysis. However, quitting self-efficacy was not identified as an important factor of smoking cessation compared with other factors such as attitude of THPA. Nevertheless, a previous study identified quitting self-efficacy as a more important factor than workplace smoking restrictions of THPA in predicting quitting smoking in smokers (Abdullah, Driezen, Quah, Nargis, & Fong, 2015). Quitting self-efficacy emphasized the confidence of smokers in their ability to abstain from smoking in high-risk situations (Etter et al., 2000). It is possible that hardcore smokers did not intend to quit smoking due to deep-rooted thoughts and behaviors regarding smoking rather than a lack of confidence in their ability to abstain from smoking in high-risk situations.

Age, years of education, and total number of years smoked were not significantly correlated with smoking cessation among hardcore smokers. Previous studies indicated that age, years, and smoked in life are not significantly associated with being hardcore smokers (Yang, Liu, Wang, & Jia, 2016). Less studies were examined between those factors and quitting smoking.

We found that social smoking motive was not significantly correlated with smoking cessation. Few studies were examined between social smoking motivate and quitting smoking. C. L. Huang et al. (2017) indicated that social smoking improves social confidence and connection. However, it is possible that individuals who smoke as adolescents or in early adulthood may feel that smoking assists them to make friends or feel part of a group, while those who smoke as adults feel like a societal outcast such as many negative commons about their smoking due to the THPA. Thus, social smoking motives may not influence resistance to smoking cessation among hardcore smokers in this study. However, further research is necessary to confirm this hypothesis.

The group with no smoking cessation experience had greater nicotine dependence than did the group with cessation experience. This result is consistent with the findings of Bommele et al. (2015). Furthermore, greater nicotine dependence was correlated with a lower likelihood of successful smoking cessation (Koks et al., 2019). However, few studies have examined the relative association between nicotine dependence and smoking cessation among hardcore smokers. We found that nicotine dependence was significantly correlated with smoking cessation prior to the inclusion of other variables in the logistic regression model. After controlling for other variables, improving attitudes regarding the THPA was significantly associated with a higher rate of smoking cessation among hardcore smokers. However, decreasing nicotine dependence become not significant. The results suggested that creating a culture around positive attitudes towards the THPA can assist in increasing the rate of smoking cessation.

### Limitations

The findings of this study should be interpreted with consideration for its limitations. First, a cross-sectional design was used. Thus, causal relationships among variables could not be inferred. Future research should, therefore, consider using longitudinal or experimental study designs to examine the causal relationship between factors and quitting smoking in hardcore smokers. Second, the sample was selected from southern Taiwan only using purposive sampling, which limited the generalizability of the findings to hardcore smokers of other areas and countries. Thus, studies involving hardcore smokers in other areas or countries are required. Third, we used a self-edited scale to assess attitudes towards the THPA, due to an absence of relevant, appropriate instruments in this regard. Nurses and researchers may, therefore, consider developing and validating such scales in future studies. Finally, hardcore smokers who were Taiwanese Asians and Taiwanese immigrants may have different factors in smoking cessation due to social culture. Further studies may focus on Taiwan immigrate.

## Conclusions

This is one of the few studies that examined the factors associated with attempt for smoking cessation among hardcore smokers. Attitudes towards the THPA were a particularly important factor contributing to attempt for smoking cessation among hardcore smokers. The finding highlighted the need to consider attitude towards the THPA as a public issue among hardcore smokers. Nurses could cooperate with smoking cessation coaches to teach correct knowledge regarding THPA for enhancing positive attitudes regarding THPA. However, Asian-Pacific Islanders who were living abroad need to abide by THPA in their country. Many countries have similar practice and policy about smoking/smoking cessation as Taiwan. For example, they sell cigarettes, but they also encourage people to stop smoking. Given this caveat, it is advised that nurses and other health clinicians promote health education about smoking cessation. Thus, the study findings from our Taiwanese populations may be applied to other Asian-Pacific Islanders overseas. In addition, the policy relating to positive attitudes regarding THPA must be clearly written and enforced in smoking cessation clinic. Government staff also need to announce importance of enforcing THPA and stopping smoking.

## Acknowledgments

The authors acknowledge the funding support by Ministry of Science and Technology.

## Declaration of Conflicting Interests

The authors declare no conflicts of interest with respect to the work, authorship, and/or publication of this article.

## Funding

This work was supported by Ministry of Science and Technology [grant numbers: 103-2314-B-309-002-MY3].
